# A Novel Photopharmacological Tool: Dual-Step Luminescence for Biological Tissue Penetration of Light and the Selective Activation of Photodrugs

**DOI:** 10.3390/ijms24119404

**Published:** 2023-05-28

**Authors:** Amador Menéndez-Velázquez, Ana Belén García-Delgado

**Affiliations:** Photoactive Materials Research Unit, IDONIAL Technology Center, 33417 Avilés, Asturias, Spain; ana.delgado@idonial.com

**Keywords:** photopharmacology, tissue penetration of light, drug activation by light, luminescence, spectral conversion, up-conversion, down-shifting

## Abstract

Conventional pharmacology lacks spatial and temporal selectivity in terms of drug action. This leads to unwanted side effects, such as damage to healthy cells, as well as other less obvious effects, such as environmental toxicity and the acquisition of resistance to drugs, especially antibiotics, by pathogenic microorganisms. Photopharmacology, based on the selective activation of drugs by light, can contribute to alleviating this serious problem. However, many of these photodrugs are activated by light in the UV–visible spectral range, which does not propagate through biological tissues. In this article, to overcome this problem, we propose a dual-spectral conversion technique, which simultaneously makes use of up-conversion (using rare earth elements) and down-shifting (using organic materials) techniques in order to modify the spectrum of light. Near-infrared light (980 nm), which penetrates tissue fairly well, can provide a “remote control” for drug activation. Once near-IR light is inside the body, it is up-converted to the UV–visible spectral range. Subsequently, this radiation is down-shifted in order to accurately adjust to the excitation wavelengths of light which can selectively activate hypothetical and specific photodrugs. In summary, this article presents, for the first time, a “dual tunable light source” which can penetrate into the human body and deliver light of specific wavelengths; thus, it can overcome one of the main limitations of photopharmacology. It opens up promising possibilities for the moving of photodrugs from the laboratory to the clinic.

## 1. Introduction

Photopharmacology [1,2,3] is an emerging field that involves the use of light to control the activity of drugs or compounds within the body. This approach has the potential to revolutionize the way we treat diseases because it allows for the precise, targeted delivery of drugs to specific areas of the body at precise times.

In this introduction, we briefly discuss the problems associated with conventional pharmacology (Section 1.1) and then the potential of photopharmacology to address these and other problems (Section 1.2). However, despite its great potential (and its proven effectiveness for “in vitro” systems), photopharmacology faces numerous barriers before it can move from the laboratory to the clinic. We analyze these limitations and describe a new and promising photopharmacological tool (Section 1.3), which is the main subject of this article.

### 1.1. Limitations of Conventional Pharmacology

Pharmacology [4,5] seeks to cure diseases and alleviate symptoms by administering drugs to the patient. It plays a crucial role in contemporary society, significantly contributing to increasing the life expectancy and the well-being of people. However, while drugs can provide significant benefits in treating diseases and conditions, they also have limitations and can produce side effects [6,7,8,9,10,11]. The World Health Organization points to two major problems and challenges in medicine: chemotherapy and bacterial resistance to antibiotics. These problems are linked to the limitations and side effects of conventional pharmacology. 

In conventional chemotherapy for cancer treatment, the drugs indiscriminately attack both cancer cells and healthy cells [12,13]. The reason for this is that chemotherapy drugs usually work by impairing mitosis in cancer cells, but they can also affect normal cells that divide quickly. This lack of spatial selectivity is what can cause some of the side effects of chemotherapy, such as hair loss and anemia.

Usually, to minimize collateral damage, conventional chemotherapy tends to reduce the optimal dose levels of the drug, making it less effective when treating cancer. Additionally, approximately 85% of anticancer drugs, some of which are potentially very effective, are clinically discarded to avoid the mentioned side effects. 

The problem of the lack of spatial (and temporal) selectivity presents adverse effects beyond the patient. When leaving the human body, the drug continues to be active and can cause environmental toxicity [14,15,16,17,18,19]. Environmental toxicity refers to the harmful effects that drugs and other chemicals can have on the environment, including the air, water, soil, and wildlife. Some drugs can be particularly harmful to the environment due to their persistence, or ability to bioaccumulate in living organisms.

Additionally, the prolonged exposure of viruses and bacteria to these drugs, both inside and outside the body, leads to a serious problem in terms of the drug resistance of pathogens [20,21,22]. Drug-resistant microorganisms can cause infections that are difficult or impossible to treat, leading to increased morbidity, mortality, and healthcare costs. This is especially serious in the case of bacteria because the prolonged exposure of bacteria to antibiotics leads to the creation of antibiotic-resistant bacterial strains. Drug resistance, especially antibiotic resistance, is a significant global public health issue, and efforts are being made to prevent and control its spread [23,24]. 

All of these side effects, which are caused to a large extent by the lack of spatial and temporal selectivity, require a new and different pharmacological approach. Photopharmacology emerges as a promising and hopeful tool to face these limitations linked to conventional pharmacology.

### 1.2. Photopharmacology: Light as a Pharmacological Switch

Photopharmacology [2,25,26,27,28,29] is based on the activation of drugs by light. When ingesting a photodrug, even if it is distributed throughout different parts of the body, it will only be activated in the precise area of the human body that is being illuminated. Therefore, spatial selectivity is achieved, which is especially useful in the case of tumors or localized pathologies. With some types of photodrugs, it is also possible to use light to restrict their pharmacological activity for a certain period of time rather than indefinitely. In this case, we achieve temporal selectivity in addition to spatial selectivity. This temporal selectivity is only possible with reversible photodrugs.

Photodrugs can be classified into two categories: reversible and irreversible. In the case of irreversible photodrugs, one possibility involves the use of molecular cages (photocages) [30] that contain the drug. Due to the effect of light, the “molecular cage” can be broken in a process known as photolysis. The drug is then released with its full pharmacological potential. However, in this case, it is not possible to return the active drug to a state of pharmacological inactivity.

Regarding reversible photodrugs, these drugs undergo a molecular change under the action of light and are able to return to their original state when irradiated with light of another wavelength or by thermal relaxation. These drugs are designed in such a way that only one of the possible molecular states has pharmacological activity. One common type of reversible photodrug is a photoswitchable molecule [31,32,33]. Photoswitchable molecules can exist in two different states. By attaching a photoswitchable group to a drug molecule, the drug can be switched between an active and an inactive state by exposure to light of different wavelengths.

We now consider the different effects of these two types of drugs. In the case of irreversible drugs, they can travel through the body without any pharmacological action until they reach the place where the focus of the disease is located, at which time they are activated by light. Therefore, in that initial journey, the drug does not cause unwanted collateral damage. However, during the subsequent journey through the body until it is eliminated from it, the drug can cause damage to healthy cells. Additionally, once removed, it can contribute to the mentioned environmental toxicity and drug resistance.

In the case of reversible photodrugs, their action is restricted both spatially and temporarily; thus, collateral damage to either the patient’s body or the environment is avoided. That is why this type of drug arouses great interest in the field of health, and there is now an active field of research around it.

### 1.3. A New Tool for Overcoming the Main Barriers of Photopharmacology

When the discipline of photopharmacology emerged, the topic of light penetration through biological tissues was completely ignored. The research focused mainly on the design of drugs that could be activated by light. “In vitro” studies were then carried out with these photodrugs, which served to validate the potential of the technology. However, to move this technology from the laboratory to the clinic, the topic of the propagation of light in the human body needs to be considered. Many of the developed photodrugs incorporate chromophores that are activated at short wavelengths, such as those corresponding to ultraviolet and visible light. However, at many of these wavelengths, human tissue shows little or no light penetration.

Light penetration as a function of wavelength and tissue type has been widely studied and described in the scientific literature. The penetration of light is limited and conditioned by absorption and scattering due to the endogenous chromophores present in the tissues. However, there is an “optical window” in biological tissues through which light can penetrate. This window is located in the near-infrared spectral range. This fact does not mean that the tissues are totally transparent to this kind of light, but rather that the absorption of light by the tissues is small, thus allowing its penetration into the human body. This optical window is located approximately between 600 nm and 1350 nm [29]. Wavelengths below 600 nm are conditioned by the absorption of hemoglobin, and wavelengths above 1350 nm are conditioned by the absorption of water. Although the depth of light penetration depends on the type of tissue, it should be noted that the wavelengths of this optical window do not behave in the same way. For example, it has been reported that 800 nm light penetrates biological tissues much better than 630 nm light [34].

In order to have light that can penetrate human tissue and at the same time activate photodrugs, spectral conversion techniques can be used. Near-infrared light can travel through the human body. Once inside, the light can be spectrally converted to specific wavelengths in the UV–visible range for the activation of specific photodrugs. Spectral conversion techniques have already found applications in fields such as photovoltaics [35,36,37,38,39], LED lighting [40,41,42], greenhouses [43,44], ophthalmology [45], anti-counterfeiting [46,47,48], etc. The photopharmacology sector could also benefit from these spectral conversion techniques. Some up-conversion research in the biomedical sector [49,50] and specifically in the photopharmaceutical sector has already been described [51,52].

In this article, we intend to go further and make use of a dual-spectral conversion technology that combines up-conversion and down-shifting processes. The excitation source is a 980 nm laser, a wavelength that can penetrate biological tissues. Making use of rare earth luminescent materials, this 980 nm radiation was up-converted to the UV–visible spectral range. By making use of organic luminescent materials, a significant fraction of this UV–visible radiation was then down-shifted to a specific UV–visible range in order to adjust the wavelengths of the emitted light to those of the specific drug’s spectral response.

The reviews by Roy Weinstain et al. [53] and Michael M. Lerch [54] et al. describe the range of spectral responses exhibited by various photodrugs and/or their potential therapeutic applications. In the research presented in this article, we did not consider any specific photodrug. This is a proof of concept to show the potential and versatility of the described technology. For the first time in the field of photopharmacology, up-conversion and down-shifting techniques are used together and sequentially. This makes it possible to achieve the double objective of the penetration of biological tissues and the delivery of light of specific wavelengths for the selective activation of photodrugs, which opens up numerous promising possibilities. The luminescent materials used were embedded in an ethylene vinyl acetate (EVA) copolymer. EVA is a copolymer with high optical performance [55,56], and it is also biocompatible [57,58]. This article also reports, for the first time, the use of EVA copolymer in the photopharmacology sector.

As mentioned, delivering light of specific wavelengths to the interior of the human body is a critical barrier when transitioning from the laboratory to clinical applications. To the best of our knowledge, there have been no “in vivo” studies conducted in humans. Brianna M. Vickerman et al. [28] reported preliminary “in vivo” studies using mice as the experimental model. In their article, they particularly highlight the challenge of UV–visible light penetration into tissues, leading them to propose photodrugs with a spectral response in the near-infrared range. However, they acknowledge that confining themselves to these near-infrared wavelengths significantly limits the scope of photopharmacology since most of the discovered photodrugs exhibit spectral responses in other areas of the spectrum than the near-infrared. Therefore, strategies such as the one proposed in this article are necessary, which further reinforces the validity and usefulness of the research presented here.

## 2. Results and Discussion

There are different spectrum manipulation technologies. We made use of luminescent materials operating as spectral converters. Through adequate selection and the combination of the luminescent materials, it is possible to absorb light of certain wavelengths and to re-emit it at different wavelengths. As mentioned, we used two spectral conversion techniques (up-conversion and down-shifting) and the luminescent materials were embedded in the EVA copolymer. As we highlighted, EVA is a copolymer with a high optical performance, and it is also biocompatible, which is why it was highly suitable for the objectives pursued in this article.

We show and discuss the results of different spectral conversions. We show the results of up-conversion using rare earth materials (Section 2.1). Then, we consider the results of down-shifting using organic materials (Section 2.2). Finally, we show and discuss the results of different kinds of molecular systems (up-converters and down-shifters) working together and sequentially (Section 2.3).

### 2.1. Up-Conversion

Up-conversion is a spectral conversion technique that is based on the absorption of photons and the subsequent re-emission of higher energy photons. That is why this process is usually called anti-Stokes. It requires the absorption of several photons so that their combined action allows the emission of higher energy photons. It is a nonlinear optical process in which, under the action of light, an electron moves from the valence band to an intermediate state of excitation to later move to a higher energy band. To prevent the electron from decaying from the intermediate band to the valence band, a high photon density is required, such as that provided by a laser. We opted for a 980 nm laser since this radiation penetrates biological tissues well. At the same time, many up-converter materials exhibit an absorption peak around 980 nm.

**Figure 1:**
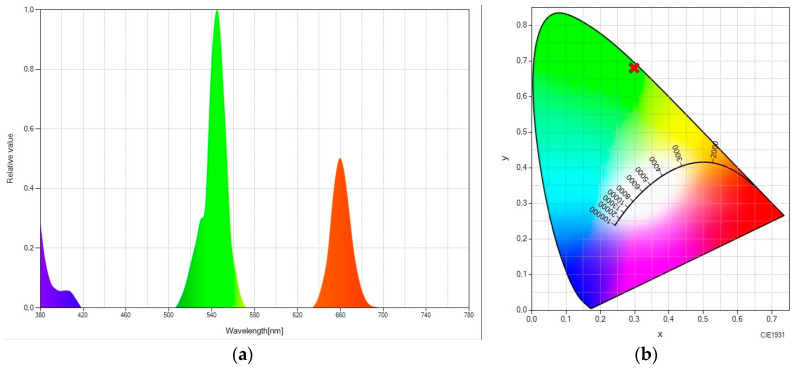
(**a**) Spectral power distribution and (**b**) CIE 1931 chromaticity diagram of “rare-earth-doped EVA” under the excitation of a 980 nm laser.

Most up-converter materials are based on rare earth materials. We made use of a rare earth material (sodium yttrium fluoride, ytterbium- and erbium-doped—NaY_0.77_ Yb_0.20_ Er_0.03_ F_4_) embedded in the EVA copolymer, which acts as a host material. This rare earth material exhibits an excitation peak at 980 nm. When irradiated with a 980 nm laser, it emits three peaks in the visible range, the most significant peak being in the green region (peaking at 545 nm). This can be seen in Figure 1a, which shows the spectral power distribution (SPD) of the rare earth material embedded in EVA after being excited at 980 nm. An SPD provides the relative radiant power emitted by a light source at different wavelengths. Figure 1b shows the CIE 1931 chromaticity diagram. This figure confirms that a large fraction of the emission falls in the green zone, with (0.2983, 0.6803) being the values of the chromatic coordinates.

### 2.2. Down-Shifting

The most conventional form of photoluminescence is referred to as down-shifting. In this process, a photon, after being absorbed by the corresponding luminescent material, is re-emitted as a lower energy photon. Therefore, in this case, the emission peak is displaced towards longer wavelengths (lower energies) with respect to the absorption peak, which is known as a Stokes shift. The energy difference between the absorbed photon and the emitted one is lost in the form of heat (non-radiative relaxation).

**Figure 2:**
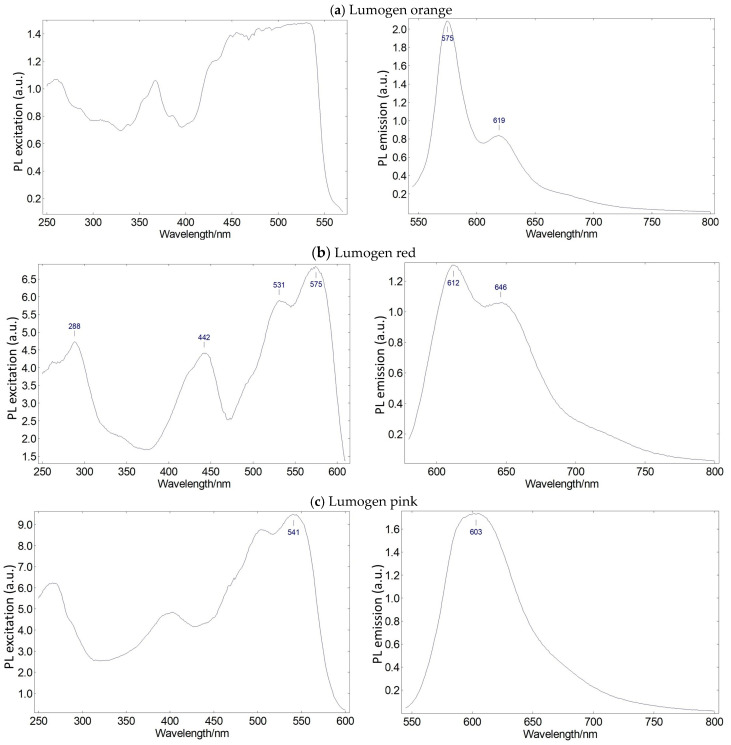
Relative photoluminescence excitation and emission spectra of (**a**) Lumogen orange embedded in EVA copolymer, (**b**) Lumogen red embedded in EVA copolymer, and (**c**) Lumogen pink embedded in EVA copolymer.

There are different types of materials that can function as down-shifters. We selected organic materials, specifically compounds from the Lumogen family, which are well known for their good performance in terms of both their optical properties and their ease of processing in solution. From the Lumogen family of materials, we selected those whose excitation spectrum overlaps with the emission peak of the green up-converter (545 nm). The selected compounds were Lumogen orange, Lumogen pink, and Lumogen red. These organic pigments were embedded in the EVA copolymer. Figure 2 shows the excitation and emission spectra of these luminescent molecular systems operating as down-shifters.

As can be seen in Figure 2, the green emission peak (545 nm) of the up-converter system is within the excitation spectral range of these three organic compounds. Therefore, all of these organic luminescent molecular systems can be excited to some extent by the rare earth materials’ luminescence. At the same time, the spectral range of emission is different in the three compounds, which guarantees versatility when designing luminescent molecular systems whose emission overlaps with the absorption of specific photodrugs.

### 2.3. Multiple Spectral Conversions Effects (Up-Conversion and Down-Shifting)

In this subsection, we consider the effects of up-conversion and down-shifting techniques operating together and sequentially. We begin by considering three spectral converters. Each spectral converter was made up of the luminescent green up-converter rare earth material and one of the three luminescent down-shifter organic molecules. These were bilayer systems, with the rare earth material in an EVA layer and the corresponding organic molecule in another EVA layer. The layer containing the luminescent rare earth material was the one closest to the 980 nm laser.

Therefore, the laser excited the luminescent rare earth material, giving rise to up-converted photons (with a green emission peak, as seen in Figure 1), a fraction of which was absorbed by the corresponding luminescent organic molecule. Then, this organic molecule re-emits the captured light according to its emission spectrum. The following figures show the SPD and chromatic coordinates of each of the three mentioned spectral converters.

**Figure 3:**
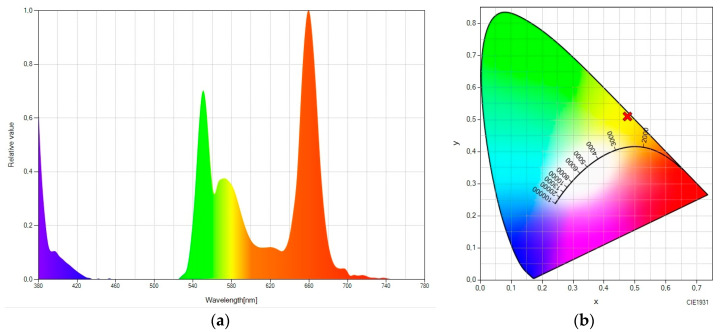
(**a**) Spectral power distribution and (**b**) CIE 1931 chromaticity diagram of “rare-earth-doped EVA + Lumogen orange-doped EVA” under the excitation of a 980 nm laser.

Let us consider the luminescent molecular system “rare earth + Lumogen orange” (Figure 3). As we have mentioned, Lumogen orange has an emission peak in the green region (575 nm), which can be seen in Figure 3a. Likewise, another considerable peak can still be seen in the green spectral range (at 545 nm), corresponding to the fraction of the up-converted green light emitted by the rare earth material, which was not absorbed by Lumogen orange. The result is an SPD with great spectral bandwidth and with chromatic coordinates in the orange zone (see Figure 3b), taking the values (0.4755, 0.5100).

**Figure 4:**
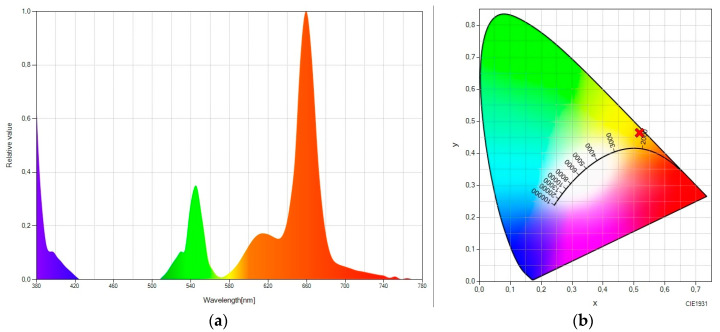
(**a**) Spectral power distribution and (**b**) CIE 1931 chromaticity diagram of “rare earth − doped EVA + Lumogen red-doped EVA” under the excitation of a 980 nm laser.

Let us now consider the luminescent molecular system “rare earth + Lumogen red” (Figure 4). Taking the pure up-converter system as a reference (Figure 1), it can be seen how a large fraction of the light is spectrally shifted to red, although there is still a residual green peak. The chromatic coordinates are close to the red area, taking the values (0.5178, 0.4643).

**Figure 5:**
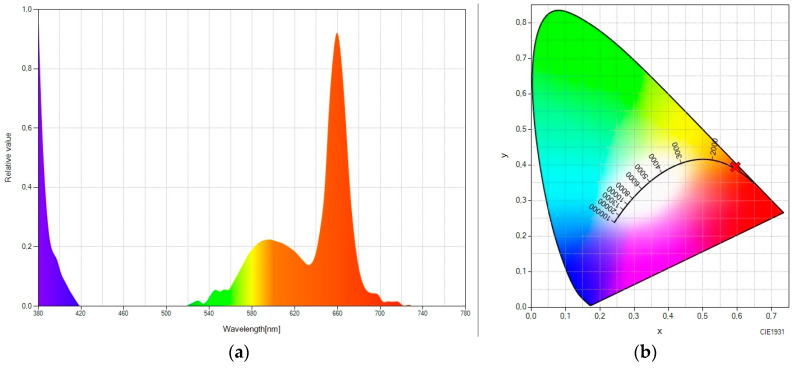
(**a**) Spectral power distribution and (**b**) CIE 1931 chromaticity diagram of “rare earth -doped EVA + Lumogen pink-doped EVA” under the excitation of a 980 nm laser.

Finally, we consider the luminescent molecular system “rare earth—Lumogen pink” (Figure 5). Again, taking the pure up-converter system as a reference (Figure 1), it can be seen (Figure 5a) how a large fraction of the light is spectrally moved to the red part of the spectrum without any emission in the green part of the spectrum. The elimination of the green peak in this spectral converter containing Lumogen pink (versus those containing Lumogen red or Lumogen orange) could be due to the fact that the excitation peak of Lumogen pink is much closer to the green emission peak of the rare earth material, which results in greater effectiveness when capturing radiation. The elimination of the green peak results in chromatic coordinates that are more shifted toward the purest red (Figure 5b), with the values (0.5942, 0.3945).

**Figure 6:**
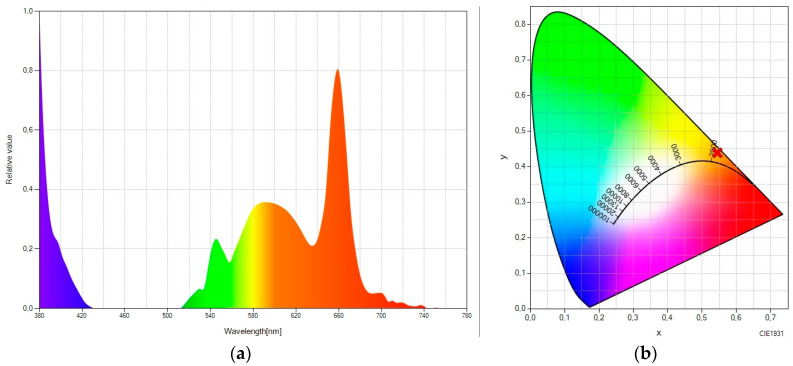
(**a**) Spectral power distribution and (**b**) CIE 1931 chromaticity diagram of “rare earth + Lumogen pink-doped EVA” (monolayer molecular system) under the excitation of a 980 nm laser.

From a practical point of view, it would be interesting to have a single luminescent EVA layer doped with both luminescent species (rare earth material and organic molecules). Taking the spectral converter shown in Figure 5 as a reference (which contained the rare earth and Lumogen pink luminescent species in different layers), we proceeded to integrate the rare earth and the Lumogen pink compounds into the same EVA layer. Figure 6 shows the corresponding SPD and chromatic coordinates.

This monolayer molecular system (with two luminescent species) worked pretty well as a spectral converter without inhibiting luminescence. Comparing Figure 5a and Figure 6a, it can be seen how the green peak is higher in the monolayer system than in the bilayer system. In this monolayer configuration, the green light emitted by the rare earth material does not encounter enough organic molecules that can intercept the path of the green light, capturing this light and converting it spectrally. Consequently, it results in less red-shifted chromatic coordinates (see Figure 6b), which take the values (0.5444, 0.4388).

In short, we showed the great tuneability and versatility of these dual systems (up-converters and down-shifters) when it comes to generating light with different spectral characteristics. The goal is to have “a tunable light source in the human body” which can activate specific photodrugs, starting from initial radiation with an emission peak at 980 nm, which can penetrate biological tissues. This article is a proof of concept, but it serves to highlight the great potential of this technology in the field of photopharmacology and to overcome its current limitations in order to move from the lab to the clinic.

## 3. Materials and Methods

### 3.1. Materials

All chemicals were used as received without further purification. Lumogen orange, Lumogen red, and Lumogen pink organic dyes were obtained from BASF (Ludwigshafen, Germany). The rare earth material used was sodium yttrium fluoride, ytterbium- and erbium-doped (NaY_0.77_ Yb_0.20_ Er_0.03_ F_4_) and was obtained from Sigma Aldrich (St. Louis, MI, USA). Dichloromethane was also obtained from Sigma Aldrich. Ethylene vinyl acetate copolymer (Evatane 33-45 PV) was obtained from Arkema (Colombes, France).

### 3.2. Luminescent Layers Preparation

The dye/rare earth-doped luminescent ethylene vinyl acetate (EVA) copolymer layers were manufactured according to the following procedure. Initially, 25 g of EVA copolymer was added to 200 mL dichloromethane and agitated at 60 °C until completely dissolved. Many of these solutions were prepared. Taking 25 g of EVA as a reference, different amounts of organic dyes (Lumogen orange, Lumogen red, or Lumogen pink) and/or rare earth material (sodium yttrium fluoride, ytterbium- and erbium-doped) were added to each solution, resulting in solutions (in the case of organic dyes) or dispersions (in the case of rare earth materials) with varying luminescent species concentrations (expressed as weight percentages with respect to EVA, which was the host material of the luminescent species). 

The solvent was then removed, resulting in luminescent EVA layers. These layers were transferred to a hot plate press where they were subjected to pressure and temperature adjustments to achieve uniformity and a specific thickness. The selected luminescent layers were as follows:

- EVA green up-converter rare earth material 5% (thickness of the layer: 0.5 mm);
- EVA Lumogen orange 0.15% (thickness of the layer: 0.8 mm);
- EVA Lumogen red 0.04% (thickness of the layer: 0.5 mm);
- EVA Lumogen pink 0.06% (thickness of the layer: 0.5 mm);
- EVA green up-conversion rare earth material 5% + Lumogen pink 0.06% (thickness of the layer: 0.5 mm).

**Figure 7:**
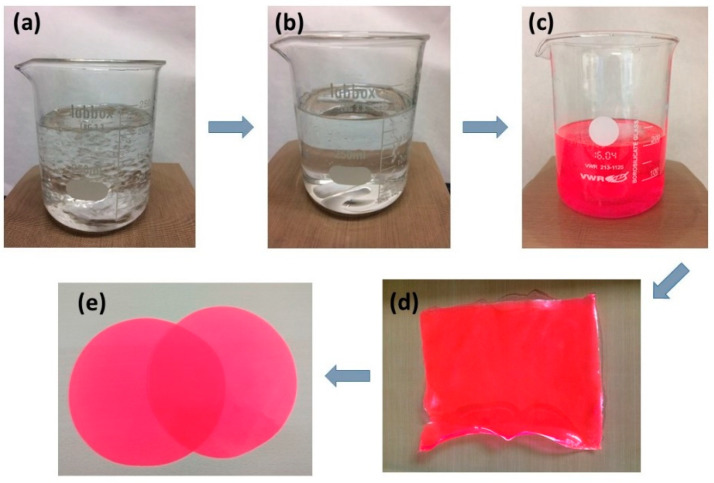
Schematic pictures illustrating the process of preparing luminescent EVA films, with Lumogen red dye serving as an example. (**a**) The dissolution process of EVA pellets in dichloromethane. (**b**) EVA pellets dissolved in dichloromethane. (**c**) EVA pellets and Lumogen red dye dissolved in dichloromethane. (**d**) Luminescent EVA layers formed by the solvent casting process. (**e**) Final luminescent EVA layers, obtained through pressing in the hot plate press.

Figure 7 illustrates the process of preparing luminescent EVA films, using Lumogen red dye as an example.

### 3.3. Optical Characterization

The measurements involving down-shifting processes were carried out using a spectrofluorometer with an excitation and emission spectral range from 200 nm to 1000 nm, UV-optimized 1800 L/mm holographic diffraction grating, and an emission detector working in the spectral range from 230 nm to 980 nm.

The measurements involving up-conversion processes (eventually followed by down-shifting processes) were carried out using a 980 nm near-infrared laser diode with a power of 400 mW. The detection of luminescence was conducted through a CRI Illuminance Meter equipped with a CMOS linear image sensor with a spectral wavelength range for detection from 380 nm to 780 nm.

## 4. Conclusions

Photopharmacology is a field of science that focuses on the use of light to control the activity of drugs in the human body. The idea is to use photosensitive molecules that can be activated by light to trigger a specific therapeutic response in the body. An inherent limitation of this technology is the penetration of light into biological tissues. In this article, to deal with this problem, we developed a set of spectral converters that captured near-infrared light (980 nm) located within the tissue penetration optical window and converted it to different wavelengths within the UV–visible spectral range.

We sequentially used two spectral conversion techniques: up-conversion (using the sodium yttrium fluoride, ytterbium- and erbium-doped—NaY_0.77_ Yb_0.20_ Er0.03 F_4_ rare earth material) and down-shifting (using the Lumogen orange, Lumogen red, or Lumogen pink organic dyes). Through the adequate selection of the down-shifter (in combination with the up-converter), it was possible to adjust the spectral range of the emitted light. To our knowledge, this was the first time that this dual technique has been used; it allowed light penetration into biological tissues and, at the same time, the delivery of light of specific wavelengths for the selective activation of specific photodrugs, making it a very valuable and promising tool in the field of photopharmacology. The mentioned luminescent species were embedded in the EVA copolymer, which guaranteed biocompatibility.

## Data Availability

The data supporting the findings of this study are available within the article.
